# Work- and hydration-related health outcomes prevalence among USA construction workers: evidence from the national survey

**DOI:** 10.3389/fpubh.2025.1721825

**Published:** 2026-01-14

**Authors:** Muinat Abolore Idris, Anna Gitter, Eva Deemer, Yue Zhang, Jingjing Gao

**Affiliations:** 1 Department of Health Promotion and Behavioral Sciences, University of Texas Health Science Center at Houston School of Public Health, Houston, TX, United States; 2 Department of Environmental and Occupational Health Sciences, University of Texas Health Science Center at Houston School of Public Health, Houston, TX, United States; 3 Department of Management, Policy and Community Health, University of Texas Health Science Center at Houston School of Public Health, Houston, TX, United States; 4 Department of Biostatistics and Data Science, University of Texas Health Science Center at Houston School of Public Health, Houston, TX, United States

**Keywords:** hydration, occupational health, construction workers, cognitive difficulty, injury, missed workdays, national survey, labor policy

## Abstract

**Background:**

Construction workers face elevated risks of heat-related illnesses, yet hydration and rest break policies remain inconsistent across regions and are not federally mandated.

**Objective:**

To evaluate the association between occupational conditions and hydration-related health outcomes among national construction workers, focusing on regional disparities and policy relevance amid increasing ambient temperatures.

**Methods:**

We analyzed 2023 National Health Interview Survey data, focusing on adults employed in the construction industry (n = 1,231) versus other industries (n = 16,241). We assessed participant self-reported back pain, diagnosed fatigue, cognitive difficulty, injury, and general health, while using regional indicators with higher temperatures, and the 2023–2024 national record-breaking heatwave, as hydration proxies. Weighted descriptive statistics, multivariate regression models, and sensitivity analyses were used to examine associations.

**Results:**

West region construction workers had the largest negative associations with back pain (β = −0.18), injury (β = −0.52), cognitive difficulty (β = −0.15), and better general health (β = −0.09), followed by the Midwest and Southern region workers for back pain and better general health. Workers’ race, sex, educational attainment, Body Mass Index (BMI), and marital status play a crucial role in workers’ reported health outcomes, with non-Hispanic Asians at higher odds of severe back pain, fatigue, and cognitive difficulty.

**Conclusion:**

Hydration access is a critical, yet underregulated factor in preventing heat-related health outcomes in occupational settings across the USA. There is an urgent need for enforceable national standards mandating water and rest breaks for construction workers, particularly in high-heat regions given recent policies that have removed water breaks in the Southern region.

## Background

Construction workers are among the most vulnerable occupational groups to extreme heat exposure and heat-related illnesses (HRIs), injuries, and fatalities. Although this occupational sector represents approximately 6% of the USA workforce, they account for over one-third of all occupational heat-related fatalities (1). Between 1992 and 2022, 986 heat-related fatalities were reported across all industries, with 334 (34%) occurring in the construction sector alone (2). These figures may likely underestimate the true burden of extreme heat exposure due to misclassification and underreporting, particularly among undocumented workers who are disproportionately represented in the construction industry. Notably, heat exposure is often exacerbated by workers’ physically demanding job tasks, prolonged outdoor work hours, inadequate access to hydration, rest breaks, and poor hydration practices, leading to dehydration.

Dehydration, a common extreme heat exposure effect, is a significant contributor to a range of adverse health outcomes, including musculoskeletal disorders [e.g., low back pain (3, 4)], cognitive impairments (5–9), general health decline (10, 11), acute and chronic kidney conditions [e.g., kidney stones (10–13), acute kidney injury (AKI) (13, 14), and chronic kidney disease (CKD) (15)]. While dehydration is a significant contributor to these health outcomes, recurrent dehydration and chronic heat stress can lead to subclinical ischemic kidney damage, which may progress to irreversible kidney dysfunction (15, 16), a health outcome prominent among outdoor workers. For instance, in 2023, ischemic heart disease was the second leading cause of fatality among USA construction workers aged 55 years and older (17). Ischemic heart disease and ischemic kidney damage conditions are both part of cardiorenal syndrome, which can persist due to poor hydration. Also, given that an estimated 52 million USA workers routinely worked outdoors, including 11.4 million in the construction industry, in 2019 (18, 19), addressing hydration practices among these workers is critical to mitigating heat-related health risks.

While there is currently no federal heat standard in the USA, the Occupational Safety and Health Administration (OSHA) proposed a heat standard called Heat Injury and Illness Prevention in Outdoor and Indoor Settings in August 2024. This is in addition to the OSHA and the National Institute for Occupational Safety and Health (NIOSH), issued recommendations, including the OSHA’s National Emphasis program, with “Water.Rest.Shade” (WRS) guideline. These recommendations promote regular consumption of water and electrolyte-rich fluids for workers; however, the implementation remains inconsistent across all occupational sectors. Prior studies have shown that hydration practices play a crucial role in mitigating heat stress and all heat-related health outcomes. Increased water and fluid intake has been found to be the most effective strategy for reducing kidney stone recurrence (20–25); electrolyte imbalance has been linked to increased AKI risk (20, 24, 26, 27); and AKI elevates cardiovascular event risks, contributing to the progression of CKD (28–30). These trends indicate the urgent need for preventive strategies that include encouraging WRS and proper hydration practices.

Despite these evidence-based findings, some state policies ban water-rest breaks, which may significantly increase outdoor workers’ vulnerability to HRIs, heat-related health outcomes (e.g., kidney damage), and even fatalities. Recent state-level legislation in Texas and Florida prohibits municipalities from enforcing mandatory water breaks to protect outdoor workers. In 2023 and 2024, Texas and Florida enacted laws—including the Texas House Bill 2127 and Florida House Bill 433—that preempt local ordinances mandating rest and water breaks for outdoor laborers, including construction workers. These policy changes occurred amid rising global ambient temperatures and intensifying heat waves, without a statewide heat protection standard, potentially increasing workers’ vulnerability. Aside from the policy potentially increasing outdoor workers’ extreme heat vulnerability, this change in policy may have a significant negative impact on the state and national economy through increased absenteeism and loss of productivity. The construction industry plays a pivotal role in the USA, contributing approximately 4.5% to the national Gross Domestic Product in 2024 (31).

While prior research has established the physiological effects of heat and dehydration among agricultural workers, with limited studies focusing on construction workers, there is a paucity of nationally representative data examining hydration-related health outcomes among construction workers. To address this gap, the present study examines the association between self-reported health outcomes related to heat exposure and hydration practice among construction workers.

## Methods

### Data sources

This study utilized publicly available data from the 2023 National Health Interview Survey (NHIS), conducted by the National Center for Health Statistics (NCHS), a division of the USA Centers for Disease Control and Prevention (CDC). The NHIS is a nationally representative, cross-sectional survey of the civilian non-institutionalized USA population. It employs a complex, multistage area probability sampling design to collect detailed information on a wide range of health topics through in-person household interviews.

### Study sample

**Figure 1:**
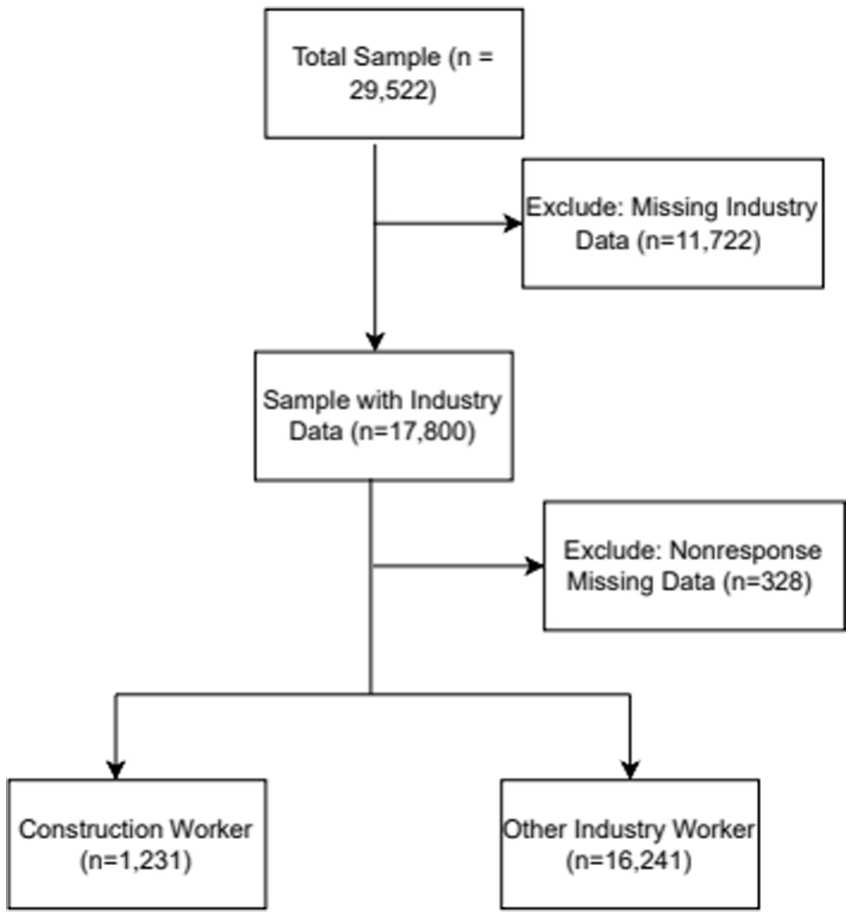
Flowchart of sample selection.

We analyzed the Adult Sample file, which includes responses from one randomly selected adult aged ≥18 years per household. The analytic sample was restricted to respondents employed in the construction industry, based on the Census Occupation and Industry Classification System, as coded by NHIS. The sampling weights, stratification, and clustering variables were applied to all analyses to generate nationally representative estimates. Key variables include cognitive difficulties, chronic fatigue symptoms, injuries resulting in days away from work (missed workdays), general health status, and back pain, as extreme heat exposure and inadequate water and electrolyte-fluid consumption can significantly influence the incidence of these hydration-related health conditions. Geographic region, demographics, employment, and health-related covariates were also analyzed. While NHIS does not directly assess participant occupational heat exposure or hydration, we constructed proxy measures based on geographic region, as (i) regions including Southern and West with higher temperatures as environmental and occupational exposure risk factors may experience greater dehydration risks, with the outdoor workers in the region mostly impacted because they may have higher heat exposure compared to their peers in the other regions. (ii) Between 2023 and 2024, the Southern region had record-breaking heatwaves and wide temperature swings, which are directly related to hydration, and Texas, alongside other states, including Oklahoma, consistently recorded extreme high temperatures over > 40 °C during this period. Figure 1 displays the analytic sample selection process. Of 29,522 respondents, we excluded participants with missing industry codes and the key variables, resulting in a final sample of 17,472 adults.

### Measured variables

- Health indicator and outcomes: Five self-reported hydration-related health outcomes were examined: back pain severity, general health status, chronic fatigue, injury, and cognitive difficulty. Participants were (i) asked how they had been bothered by back pain over the past 3 months; (ii) if they had difficulty concentrating or remembering; and (iii) if they had ever been diagnosed or still have chronic fatigue syndrome to assess participants’ back pain, cognitive difficulty, and fatigue, respectively. Participants were also asked the number of workdays missed due to injury in the past 3 months, accounting for their absenteeism due to injury, and how they perceive their health, to assess their injury and general health. Additionally, participants’ body mass index (BMI), which was calculated from self-reported weight and height, was analyzed. While self-reported measurements are subject to reporting bias, BMI remains a widely used indicator of general health and physical resilience.
- Geographic region: Region was included as a key variable to account for regional differences in the health outcomes. In accordance with the USA Census Bureau classification, the region was categorized into four categories: Northeast, Midwest, Southern, and West, which were analyzed as categorical covariates in all the adjusted models to control for geographic variation.
- Covariate factors: Included socioeconomic and demographic covariates were educational attainment, age, sex, race, marital status, and health insurance. These variables were selected given they may influence health outcome vulnerability (32–37).

### Statistical analysis

To prepare variables for analysis, multiple responses were recoded for interpretability and to align with modeling requirements. Construction workers were identified using industry classification codes, with participants coded as “1” if they reported working in the construction industry (emdindstn2_a == 4) and “0” otherwise. Health outcomes, region, and covariate variables were cleaned by removing non-substantive responses such as “Refused,” “Not ascertained,” and “Do not know.” Fatigue [ever diagnosed (cfsev_a) and current (cfsnow_a)] was recoded as binary variables, with “1” indicating any reported fatigue and “0” indicating none. Back pain was recoded to improve the ordinal interpretability of pain severity levels (paiback3m_a), which was based on the original NHIS codebook definition. Sociodemographic variables were also recoded to consolidate categories and exclude implausible or missing values. These transformations ensured consistency across variables and supported valid inference in the weighted regression models. Educational attainment was assessed using educp_a, which was categorized into five, to enhance interpretability and facilitate comparisons across education levels. This recoding was based on established conventions in public health research and ensured adequate representation within each category for regression modeling. Missing or implausible education values were excluded from adjusted models.

Descriptive analyses were conducted first to compare construction workers with all other workers. Proportions and means were compared using survey-adjusted chi-square tests and t-tests as appropriate. To examine the association between heat exposure, hydration practices, and hydration-related health outcomes, we employed different regression models based on the type of outcome variable. All outcomes were modeled as binary or ordinal based on their nature, using survey-weighted logistic-and-ordinal logistic regression models. Appropriate sampling weights (wtfa_a), strata (pstrat), and primary sampling units (ppsu) were applied to account for the NHIS complex sampling design, produce nationally representative estimates, and correct variance estimation.

Multivariate analyses were also conducted. Rao-Scott adjusted chi-square test was used to assess whether the characteristics distributions differ between construction and non-construction workers, adjusting for the survey design, and providing design-adjusted *p*-value output for group comparisons. Survey-weighted ordered logistic and logistic regression models were used to examine predictors of back pain, general health status, cognitive difficulty, injury, and fatigue, respectively, adjusting for all the covariates, region, and BMI in all models. To test robustness, we conducted four sensitivity analyses, excluding the fatigue health outcome due to a small sample size, and made changes to the reference group. First, we restricted the sample to working-age construction workers (ages 18–64) and re-estimated the logistic regression for back pain, general health, cognitive difficulty, and injury. While studies have shown that one of the effects of heat strain is mental confusion, which may arise due to cognitive difficulty, it is crucial to understand that the reported and assessed cognitive difficulty in this study was self-reported, which may not accurately mirror workers’ cognition, a limitation, making the need for cautious interpretation of our findings. Similar to the multivariate analyses, the models were adjusted for region, sociodemographic, and health indicator covariates. Adjusted odds ratios (aORs) and 95% confidence intervals (CIs) were reported for all associations. All analyses were conducted using SAS 9.4.

## Results

### Sample description

Table 1 presents the weighted descriptive characteristics. Construction workers were overwhelmingly male (89.0% vs. 11.0%, *p* < 0.001), non-Hispanic White (60.3%), with mostly high school (35.1%) and some college/associate (31.2%) educational attainment, and were more concentrated in the Southern region (41.0%). A greater proportion of construction workers were overweight (38.2%) and obese (36.4%). While general health proportion, cognitive difficulty, and fatigue patterns were broadly similar between groups, construction workers showed slightly higher rates of self-reported injuries leading to days away from work, which also accounts for workers’ absenteeism, and moderate back pain.

**Table 1:** Weighted descriptive characteristics of construction workers compared to other USA workers, NHIS 2023.

Variable	Non-construction (%)	Construction (%)	*p*-value
Weighted *N* in million	151.6 (92.1)	12.9 (7.8)	
Socioeconomic and Demographic			
Age (mean ± SE)	41.93 ± 0.15	42.65 ± 0.51	0.18
Sex			
Male	7,497 (50.2)	1,049 (89.0)	< 0.0001
Female	8,361 (49.8)	148 (11.0)	
Race			
Hispanic	2,555 (17.2)	321 (31.0)	<0.0001
Non-Hispanic White (NH White)	10,053 (61.1)	776 (60.3)	
Non-Hispanic Black (NH Black)	1730 (12.0)	67 (5.7)	
Non-Hispanic Asian (NH Asian)	1,082 (6.9)	17 (1.2)	
Other/Mixed	421 (2.8)	16 (1.6)	
Insurance			
Insured	14,780 (92.5)	998 (82.9)	<0.001
Uninsured	1,064 (7.5)	197 (17.1)	
BMI			
Underweight (<18.5 kg/m^2^)	201 (1.3)	8 (0.8)	0.002
Normal (18.5–24.9 kg/m^2^)	4,795 (30.3)	288 (24.5)	
Overweight (25.0–29.9 kg/m^2^)	5,443 (33.9)	480 (38.2)	
Obese (≥30.0 kg/m^2^)	5,419 (34.3)	421 (36.4)	
Region			
Northeast	2,433 (17.2)	169 (15.3)	0.179
Midwest	3,540 (21.2)	244 (19.7)	
Southern	5,748 (37.6)	481 (41.0)	
West	4,137 (24.0)	303 (23.9)	
Marital status			
Married	7,419 (50.7)	601 (54.9)	0.023
Not Married	8,439 (49.3)	596 (45.1)	
Education levels			
< High School	804 (6.5)	161 (16.8)	<0.0001
High School/GED	3,427 (24.1)	430 (35.1)	
Some College/Associate	4,333 (29.9)	366 (31.2)	
Bachelor’s Degree	4,412 (24.2)	196 (13.8)	
Master’s/Doctorate	2,882 (15.2)	44 (3.0)	
Health outcomes			
Cognition			
No difficulty	13,391 (84.1)	1,031 (87.3)	0.038
Some difficulty	2,231 (14.3)	154 (11.7)	
A lot of difficulty	236 (1.6)	10 (0.9)	
Workplace injury			
No	628 (61.8)	41 (51.9)	0.157
Yes	384 (38.2)	32 (48.1)	
Fatigue			
Ever Fatigued	46 (28.1)	4 (28.4)	0.984
Currently Fatigued	108 (71.8)	6 (71.6)	
General health			
Excellent	3,940 (25.4)	305 (26.4)	0.032
Very Good	6,072 (37.9)	414 (33.1)	
Good	4,410 (27.6)	356 (31.1)	
Fair	1,279 (8.0)	105 (8.0)	
Poor	157 (0.9)	17 (1.3)	
Back pain			
Not at all	3,375 (32.2)	230 (31.6)	0.97
A little	3,795 (35.9)	286 (36.8)	
Somewhere in between	1759 (17.0)	130 (16.6)	
A lot	1,559 (14.8)	112 (15.0)	

Weighted *N* (frequencies) are calculated using NHIS person-level survey weights and represent the estimated number of individuals in the USA civilian noninstitutionalized population; the values are reported in millions and rounded to one decimal place.

**Figure 2:**
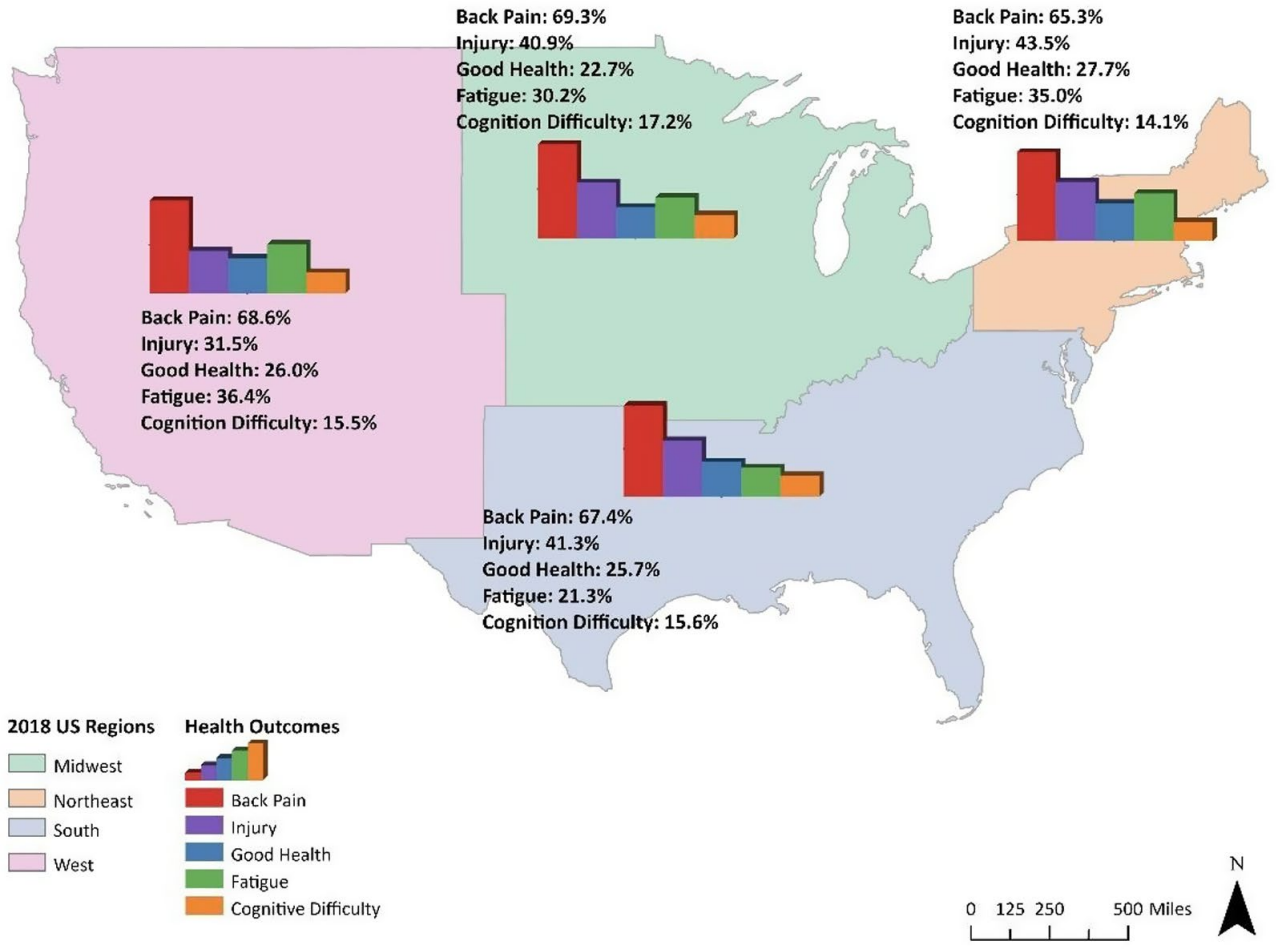
Hydration-related health burden among USA construction workers by region.

Mapping the distribution of health outcomes for only construction workers revealed a slight variation by region, with workers residing in the West reporting the highest fatigue (36.4%) and the lowest injury (31.5%) (Figure 2). The reported fatigue is a combination of participants who have ever been diagnosed with chronic fatigue syndrome and those who still have the syndrome. While good general health is consistently low across regions (less than 28%), workers reported general health was mainly moderate, with approximately 63.9–68.4% across all regions, and a relatively small proportion of poor general health (8.3–9.5%) (Supplementary Table 1). Midwest region workers reported the highest moderate general health, while the Southern region had the highest poor general health. Roughly two-thirds of construction workers across all regions are bothered by back pain, with the Northeast region reporting the lowest back pain (65.3%), and the Southern region reporting the lowest fatigue proportion (21.3%).

### Multivariable analyses

Table 2 presents the adjusted regression models examining associations between sociodemographic and hydration-related health outcomes. Older age (β = −0.02, *p* < 0.05) was significantly associated with reporting poor general health, with higher BMI [overweight (β = −0.30, *p* < 0.05) and obese (β = −1.04, *p* < 0.05)] surprisingly significantly associated with lower reporting of poor general health. Contrarily, construction workers who identified as non-Hispanic White (β = 0.12, *p* = 0.02), married (β = 0.20, *p* < 0.0001), and having higher educational attainment levels (*p* < 0.0001) were significantly associated with reporting better general health. Non-Hispanic Asians and Others/Mixed race workers had significantly higher odds of fatigue (*p* < 0.0001) and were also less likely to report better general health.

**Table 2:** Adjusted regression estimates for associations between demographic and socioeconomic characteristics and hydration-related health outcomes among USA construction workers, NHIS 2023.

Variable	Back pain β ± SE	*p*-value	General health β ± SE	*p*-value	Fatigue β ± SE	p-value	Injury *β* ± SE	*p*-value	Cognitive difficulty β ± SE	*p*-value
Age	0.0045 (0.0014)	0.001	−0.02 (0.001)	<0.0001	−0.005 (0.02)	0.721	0.0048 (0.006)	0.394	0.004 (0.002)	0.021
Sex (Ref = Female)	0.175 (0.041)	<0.0001	0.05 (0.03)	0.16	−0.446 (0.527)	0.399	−0.18 (0.16)	0.27	0.32 (0.05)	<0.0001
Race (Ref = Hispanic)										
NH White	−0.147 (0.06)	0.016	0.12 (0.05)	0.02	−0.424 (0.798)	0.596	−0.076 (0.236)	0.746	−0.38 (0.08)	<0.0001
NH Black	−0.155 (0.08)	0.081	−0.08 (0.07)	0.28	−0.898 (1.145)	0.435	0.605 (0.311)	0.052	−0.09 (0.11)	0.36
NH Asian	0.088 (0.101)	0.384	−0.20 (0.08)	0.01	12.516 (2.171)	<0.0001	0.322 (0.364)	0.376	0.41 (0.14)	0.004
Others	−0.312 (0.139)	0.024	−0.24 (0.10)	0.02	12.824 (0.971)	<0.0001	−0.275 (0.534)	0.607	−0.33 (0.17)	0.05
Insurance (Ref = No)	−0.0085 (0.084)	0.920	0.03 (0.07)	0.68	−1.103 (1.099)	0.319	0.207 (0.290)	0.476	−0.19 (0.103)	0.063
BMI (Ref = Normal)										
Underweight	0.086 (0.190)	0.653	−0.15 (0.152)	0.32	0.237 (1.553)	0.879–0.099 (0.63)		0.87	−0.16 (0.20)	0.43
Overweight	−0.138 (0.05)	0.007	−0.30 (0.05)	<0.0001	−0.377 (0.554)	0.498	0.231 (0.197)	0.24	0.08 (0.07)	0.26
Obese	−0.327 (0.05)	<0.0001	−1.04 (0.04)	<0.0001	0.565 (0.518)	0.277	0.116 (0.199)	0.56	−0.17 (0.06)	0.007
Region (Ref = Northeast)										
Midwest	−0.131 (0.064)	0.043	−0.13 (0.06)	0.023	0.142 (0.589)	0.809	−0.110 (0.267)	0.679	−0.14 (0.08)	0.09
Southern	−0.136 (0.061)	0.027	−0.04 (0.05)	0.45	0.604 (0.525)	0.254	−0.217 (0.254)	0.395	−0.09 (0.08)	0.23
West	−0.181 (0.063)	0.0045	−0.09 (0.06)	0.09	−0.111 (0.671)	0.869	−0.515 (0.272)	0.05	−0.15 (0.09)	0.09
Marital Status (Ref = Not Married)	−0.023 (0.04)	0.578	0.20 (0.04)	<0.0001	−0.346 (0.448)	0.442	−0.304 (0.164)	0.065	0.37 (0.05)	<0.0001
Education (Ref = < HS)										
HS/GED	0.0049 (0.108)	0.964	0.34 (0.08)	<0.0001	0.547 (1.344)	0.684	−0.398 (0.402)	0.323	0.21 (0.10)	0.04
Some Colleges/AA	0.0335 (0.104)	0.747	0.53 (0.09)	<0.0001	0.321 (1.316)	0.808	−0.463 (0.405)	0.254	0.18 (0.11)	0.11
BA/BS	0.226 (0.113)	0.0449	0.85 (0.09)	<0.0001	0.003 (1.247)	0.998	−0.838 (0.412)	0.043	0.49 (0.11)	<0.0001
Graduates	0.3099 (0.1167)	0.008	1.00 (0.09)	<0.0001	−0.406 (1.334)	0.782	−1.247 (0.415)	0.003	0.70 (0.12)	<0.0001
Observations	17,055		17,055		164		17,055		17,055	

Coefficients are reported with standard errors in parentheses. Statistical significance: *p < 0.05, **p < 0.01, ***p < 0.001, *p* < 0.0001. NH, non-Hispanic; HS, High school; Graduates, master’s and doctoral degrees; Ref, reference group.

With injury models, non-Hispanic Black workers showed a positive association with reporting injury (β = 0.61, *p* = 0.05), indicating they were more likely to self-report injury resulting in missed workdays compared to the Hispanic workers. This noticeable association could be due to potential confounders, including immigration status, which is beyond the scope of our study, as it is well-known that construction workers are mostly undocumented workers, who may be fearful of reporting their injuries, which may result in under-reporting workplace injuries rather than a difference in injury status by race. Being married and having a college education or higher were both associated with a reduced likelihood of reporting injuries resulting in missed workdays and absenteeism. The back pain models show that males (β = 0.18, *p* < 0.0001), older workers (β = 0.004, *p* = 0.001), and those with higher levels of education (bachelor’s and graduate degrees) were positively associated with higher reporting of severe back pain. Compared to other races, non-Hispanic Asian workers showed a positive association with slightly higher self-reports of back pain; the association does not provide strong evidence of a higher likelihood, because the result was not statistically significant (β = 0.09, *p* > 0.05). Surprisingly, workers who were overweight or obese were significantly less likely to report severe back pain, with underweight workers at increased risk of reporting back pain. This is an uncommon finding with most previous studies that identified a history of back pain as a significant contributor, but recently, BMI has been identified as one of the new global modifiable risk factors for low back pain.

The cognitive difficulty models show that older age (β = 0.004, *p* < 0.05) is associated with a slight increase in self-reported cognitive difficulty. While construction workers who identified as male (β = 0.32, *p* < 0.0001), non-Hispanic Asian (β = 0.41, *p* < 0.01), and married (β = 0.37, p < 0.0001) had significantly lower odds of experiencing cognitive difficulty. Educational attainment showed a strong inverse relationship with cognitive difficulty, with higher education—particularly (bachelor’s: β = 0.49, *p* < 0.0001, and graduate degrees: β = 0.7, *p* < 0.0001) were significantly associated with better cognitive functioning and lower odds of reporting cognitive impairment. Conversely, workers who were obese (β = −0.17, *p* < 0.01) and those who identify as non-Hispanic White (β = −0.38, *p* < 0.0001) or mixed race (β = −0.33, *p* = 0.05) reported significantly lower cognitive difficulties. Lastly, construction workers who identified as male (β = −0.45) reported lower fatigue levels in the fatigue models, although the effect was moderate and not statistically significant. Workers who are overweight (β = −0.38) also showed a weak negative association with fatigue, while those who are obese (β = 0.57) reported higher fatigue levels. Compared to the Hispanic, workers who identified as non-Hispanic White (β = −0.43) and non-Hispanic Black workers (β = −0.90) were negatively associated with fatigue, although the non-Hispanic Black estimate may be unstable. In contrast, the non-Hispanic Asian (β = 12.52) and mixed race (β = 12.82) shows substantial positive associations with fatigue, suggesting substantial reporting differences. While being married (β = −0.35) was associated with slightly lower fatigue, age (β = −0.005) showed no meaningful association.

Having insurance was associated with lower levels of self-reported fatigue (β = −1.10), although this effect was not statistically significant (*p* > 0.05). Insurance status showed no significant associations with cognitive difficulty, back pain, general health, or injury. Variation differences were observed across regions for the health outcomes. Compared to the Northeast region, workers residing in the West region consistently showed the largest negative associations with back pain (β = −0.18), injury (β = −0.52), cognitive difficulty (β = −0.15), and better general health (β = −0.09). The Midwest and Southern region workers also showed negative associations with back pain (β = −0.13 and β = −0.14, respectively) and better general health (β = −0.13 and β = −0.04), although the effects were smaller. The regional differences were minimal for fatigue, with coefficients close to zero and no significance.

### Sensitivity analyses

The sensitivity analyses restricted to working-aged 18–64 years supported the robustness of these findings. The age-restricted model show that increasing age was associated with increased odds of self-reporting back pain (aOR = 1.31, 95% CI: 1.15–1.15) and fair/poor general health (aOR = 1.59, 95% CI: 1.42–1.78), but lower odds of cognitive difficulty (aOR = 0.80, 95% CI: 0.69–0.94) (Table 3). However, this result should be interpreted with caution, as increasing age correlates with increased dose of physical exertion, and back pain is a cumulative strain-type injury, also, increasing age generally correlates with poorer health, although the relationship is complex and influenced by biological, social, and behavioral factors.

**Table 3:** Sensitivity analysis: regression results with age restriction (age 18–64 years).

Variable	Back pain	General health	Cognitive difficulty	Workplace injury
	(aOR [95% CI])	(aOR [95% CI])	(aOR [95% CI])	(aOR [95% CI])
Age	1.31 [1.148, 1.149]	1.59 [1.42, 1.78]**	0.80 [0.69, 0.94]*	0.80 [0.47, 1.35]
Sex: (Male)	1.07 [0.97, 1.18]	0.96 [0.89, 1.04]	1.37 [1.24, 1.53]**	1.21 [0.88, 1.66]
Race: (NH White)				
Hispanic	0.83 [0.72, 0.95]*	0.84 [0.75, 0.94]*	0.71 [0.61, 0.82]**	1.07 [0.67, 1.69]
NH Black	0.88 [0.74, 1.04]	0.72 [0.63, 0.82]**	0.76 [0.63, 0.91]*	2.00 [1.21, 3.31]
NH Asian	0.80 [0.65, 0.98]*	0.63 [0.53, 0.75]**	0.46 [0.36, 0.59]**	1.48 [0.78, 2.83]*
Others/Mixed	1.17 [0.86, 1.59]	0.73 [0.58, 0.92]*	0.99 [0.73, 1.35]	0.81 [0.30, 2.20]
Insurance:(Yes)	0.93 [0.76, 1.13]	1.00 [0.87, 1.16]	0.84 [0.69, 1.03]	0.83 [0.47, 1.47]
BMI: (Overweight)				
Underweight	0.75 [0.49, 1.15]	1.47 [1.01, 2.15]*	1.34 [0.90. 1.99]	0.70 [0.20, 2.39]
Normal	0.89 [0.79, 1.00]*	1.32 [1.20, 1.46]**	1.10 [0.97, 1.26]	0.78 [0.53, 1.14]
Obese	1.13 [1.01, 1.27]*	0.48 [0.44, 0.52]**	1.29 [1.14, 1.46]**	0.89 [0.61, 1.29]
Region:(Midwest)				
Northeast	0.89 [0.76, 1.04]	1.09 [0.95, 1.25]	0.86 [0.73, 1.02]	1.13 [0.67, 1.91]*
Southern	0.96 [0.85, 1.08]	1.02 [0.92, 1.14]	0.95 [0.82, 1.08]	0.89 [0.60, 1.33]
West	1.05 [0.93, 1.20]	0.97 [0.86, 1.09]	1.00 [0.86, 1.16]	0.67 [0.43, 1.04]
Marital status: (Married)	1.00 [0.91, 1.09]	0.98 [0.91, 1.07]	1.51 [1.36, 1.67]**	1.29 [0.94, 1.77]
Education:(BA/BS)				
HS	1.06 [0.82, 1.38]	0.36 [0.30, 0.43]**	1.59 [1.27, 1.99]**	2.30 [1.01, 5.21]
HS/GED	1.18 [1.03, 1.36]	0.55 [0.49, 0.61]**	1.30 [1.12, 1.50]*	1.55 [0.99, 2.42]
Some College/AA	1.09 [0.96, 1.23]	0.68 [0.61, 0.75]**	1.34 [1.17, 1.55]**	1.45 [0.97, 2.15]
Graduate	0.94 [0.76, 1.09]	1.08 [0.96, 1.22]	0.80 [0.67, 0.94]*	0.66 [0.39, 1.13]

aOR, adjusted odds ratio. Statistical significance: *p < 0.05, **p < 0.001.

Race was consistently associated with better-reported general health across several domains compared with the non-Hispanic White workers. This could be due to multiple potential confounding factors, including cultural differences in health perception, immigration status, and chronic disease burden. Hispanic, non-Hispanic Black, and non-Hispanic Asian workers generally had lower odds of reporting back pain, poor general health, and cognitive difficulty, though non-Hispanic Black workers were two times more likely to report injury (aOR = 2.00, 95% CI: 1.21–3.31), compared with non-Hispanic White. Obesity was associated with increased odds of back pain (aOR = 1.13, 95% CI: 1.01–1.27) and cognitive difficulty (aOR = 1.29, 95% CI: 1.14–1.46), while underweight and normal-weight workers showed mixed associations, with those who are underweight related to higher odds associated with poor general health, and obese workers having lower odds of reporting poor general health. This association suggests a nonlinear relationship between BMI and workers’ general health when compared with the multivariate findings. Educational attainment showed strong and consistent associations across all the health outcomes assessed. Compared with college graduates, workers with a high school, GED, or some college had substantially higher odds of reporting cognitive difficulty, back pain, and injury, underscoring the role of educational disparities in measured health outcomes among USA working-age adults.

While insurance status showed no significant association in the models, regional differences remain consistent with some of the multivariate findings. Compared with workers in the Midwest, Northeast workers had lower odds of back pain (aOR = 0.89; 95% CI: 0.76–1.04) and cognitive difficulty (aOR = 0.86; 95% CI: 0.73–1.02), while West workers had lower odds of injury (aOR = 0.67; 95% CI: 0.43–1.04). In contrast to the multivariate findings, the sensitivity analysis showed that workers living in the Northeast had slightly higher odds of reporting poor general health (aOR = 1.09, 95% CI: 0.95–1.25). However, this association was not statistically significant. Notably, compared to the Midwest, the Southern region showed no statistical significance across the four health outcomes, which suggests a similar injury and general health outcome profile between construction workers in the Southern and Midwestern regions. The Southern region, one of the regions with the highest number of undocumented construction workers, with about 50% in Texas and 38% in Florida, has an immigration status that could potentially hinder the workers from reporting injuries, which may lead to under-reporting of injuries, which could confound the NHIS national injury data report.

## Discussion

This study provides new nationally representative evidence on the hydration-related health risks faced by USA construction workers, which may be related to workers’ heat exposure and hydration practice. We identified several sociodemographic and regional factors associated with the adverse hydration-related health outcomes, with notable patterns emerging across the measured outcomes, which are explicitly discussed below.

### General health

In the multivariate analyses, increasing age was consistently associated with poorer general health, with sensitivity analyses showing that older workers had 59% higher odds of self-reporting poor health. This age-related pattern is consistent with a systematic meta-analysis that reported that the prevalence of CKD significantly increases with age (38). CKD is a well-known health condition that can be exacerbated by repeated heat exposure and dehydration. Marital status emerged as a protective factor for general health, with married construction workers reporting better health. This finding is consistent with prior studies that found that unmarried individuals are more likely to report poor sleep and poorer general health (39, 40). BMI was another significant factor influencing workers’ perceived health, which was strongly associated with their general health. Interestingly, overweight and obese workers had lower odds of reporting poor health compared to those with normal BMI. While this finding may appear unexpected given the well-established risks associated with obesity, the finding may reflect context-specific dynamics, including reverse causality association, where workers with chronic health conditions may experience weight loss and fall into the normal or underweight categories. The sensitivity analysis further revealed that underweight workers had the highest odds of reporting poor general health, followed by workers with normal BMI, while obese workers had the lowest odds. Nearly half of underweight workers (47%) and one-third of normal-weight workers (32%) reported good health, while prevalence was notably higher among overweight and obese (52%) workers. These results suggest that lower BMI may be linked to increased vulnerability to poor general health in physically demanding jobs, as hydration may play a significant role in the pattern observed. A systematic and meta-analysis has linked normal-weight obesity (normal BMI with high body fat) to increased cardiometabolic risk (41). Also, Jacques et al. (42) reported that underhydration is associated with a higher risk of poor lipid profiles and inflammation. However, with construction workers and construction industry settings, where heat exposure and fluid loss are common, underweight and normal-weight workers may be more susceptible to dehydration-related fatigue, which may influence their general health. Also, the combination of extreme heat exposure, high physical job demands/activity, and inadequate hydration may amplify these risks.

### Back pain

This study identified several sociodemographic factors associated with self-reported pain. Male workers have 19% increased odds of back pain, a consistent finding with Fatoye et al.’s (43) systematic review. Contrary to these, Bento et al. (44) reported a higher back pain prevalence among women. However, despite these reported differences, Bento et al. and Fatoye et al. both identified occupational factors, including physical workload, activity intensity; lifestyle factors (e.g., BMI); and demographics (e.g., age and race), as key determinants. These determinants are also relevant factors to construction workers because they are exposed to strenuous physical activity and heat. Secondly, age was consistently associated with increased odds of back pain, with older workers at 31% higher odds. A finding that reflects Jia et al.’s (45) study, which found that older age professional drivers, repetitive movements, static postures, and insufficient rest breaks contributed to low back pain, indicating the potential modifying role hydration may play. Also, BMI showed a nonlinear relationship with back pain, with the multivariate analyses results indicating that overweight and obese workers had lower odds of experiencing back pain compared to workers with normal BMI. Contrary to this, the sensitivity analysis showed that obese workers had 13% higher odds of experiencing back pain relative to overweight workers. This is consistent with prior studies that linked higher BMI with musculoskeletal strain and inflammation (46, 47), which can lead to increased back pain, suggesting that while moderate excess weight may look protective, being obese may increase the prevalence of back pain risk among construction workers. Additionally, while educational attainment was positively associated with back pain in the multivariate model, with higher odds among workers with BA/BS and graduate degrees compared to workers with less than a high school diploma, the sensitivity analysis showed that workers with HS/GED had significantly higher odds of back pain compared to those with a BA/BS. This finding suggests that back pain may be influenced by both educational attainment and occupational exposure.

These findings underscore the complex interplay between social determinants of health and back pain, highlighting the need for and importance of targeted occupational health interventions that take into consideration workers’ sex, age, race, educational level, body composition, and possibly back pain history in addressing occupational health disparities and future back pain issues. However, given the intensive, physically demanding activity construction work entails, construction workers’ sex, age, educational level, race, and BMI, combined with inadequate hydration and insufficient fluid intake, may exacerbate musculoskeletal risks, increasing workers’ susceptibility to back pain.

### Cognitive difficulty

Cognitive difficulty among construction workers varied by sex, age, education, BMI, and race, underscoring the influence of both social and health determinants. Male workers reported higher odds of experiencing cognitive difficulty, although this effect differed by race. Hispanic, non-Hispanic Black, and non-Hispanic Asian workers reported lower odds than the non-Hispanic White workers. These sex and racial differences may be due to occupational exposures or cultural differences, considering that about 90% of USA construction workers are male and mostly non-Hispanic White (approximately 57.5%). However, our finding is consistent with a prior study that found that sex- and race-based cognitive differences are not uniform across racial groups (48). The observed age patterns association was mixed, with the regression model showing that older workers generally reported greater cognitive difficulty, while the sensitivity analyses indicate a reduced odds for the restricted work aged (18–64 years), possibly suggesting a protective effect of age and reflecting the benefits of work experience. Consistent with prior literature, older age is independently associated with lower cognitive scores (49). Also, educational attainment level showed a clear and strong gradient; construction workers holding a BA/BS degree had 63% lower odds of experiencing cognitive difficulty, and workers with graduate degrees had over 100% lower odds compared to workers with less than a high school education. The sensitivity analyses confirmed these patterns, which is also supported by a prior study that higher education, particularly advanced degrees, provides substantial cognitive protection (50). Furthermore, BMI was also identified as a significant factor, although the relationship was more nuanced, with obese workers having lower odds of self-reporting cognitive difficulty when compared with normal BMI workers, but higher odds when the overweight group was the reference group, making the association complex. This is consistent with the “obesity paradox,” where obesity may increase risk compared to being overweight but appears protective compared to normal weight (51, 52).

Notably, occupational exposures to heat, which can increase the risk of heat stress, dehydration, and fatigue, are common among construction workers. This may compound workers’ risks by impairing their memory, attention, and decision-making. These findings highlight the need for workplace interventions—including hydration promotion and health management strategies—to protect cognitive function and reduce disparities among construction workers.

### Injury

Injury leading to missed workdays was strongly associated with workers’ education and race. Construction workers with only a high school diploma had more than twice the odds of injury compared with workers with a bachelor’s degree, while holding a graduate degree indicates the lowest risk, which aligns with prior studies (53–55). Also, a noticeable racial disparity was significantly observed, with non-Hispanic Black workers reporting higher injury odds than Hispanic and non-Hispanic White workers, even after adjusting for education, which is consistent with Taylor et al. (55), who reported disproportionate traumatic injury risk among minority race workers. These higher odds could be due to potential confounders, including immigration status and cultural perception. Although the findings for Hispanic and non-Hispanic Asian workers were not statistically significant, the direction of the estimates suggests potential inequities. With the construction workforce population, holding a high school diploma or below may reduce workers’ awareness or adoption of protective behaviors—including proper hydration, thereby heightening their vulnerability to heat-related fatigue and injury.

Our results reinforce educational level as a protective factor and highlight persistent racial disparities in occupational health, indicating and suggesting the need for targeted interventions, including culturally tailored hydration promotion and equitable workplace protections as crucial strategies to mitigate injury risk, improve workers’ resilience working in hot environments, and advance health equity among construction workers.

### Fatigue

Construction workers ever diagnosed and still having fatigue were shaped by workers’ educational level, race, insurance status, and BMI, with higher education attainment showing a protective trend, which is consistent with prior studies that educational attainment improves/increases access to resources and health practices that help mitigate occupational risks, including hydration awareness (54, 55). While workers with a high school diploma or less may limit workers’ awareness of hydration needs and rest break rights, increasing workers’ vulnerability to heat-related fatigue. Racial disparities were notable in our study, with non-Hispanic Asian and Mixed/Other workers reporting higher odds of reported fatigue, although the small sample sizes [n = 164] might affect the observed association. However, broader inequities remain relevant, as Hispanic workers—who represent one-third of the USA construction workforce face disproportionate heat exposure, limited access to rest-shade, and reduced workplace protections, which may increase dehydration and fatigue risks among construction workers. Obesity was found to be associated with higher odds of workers ever diagnosed or still having fatigue, while males, insured, and married workers showed a moderately lower fatigue risk. These findings underscore fatigue as a multifactorial risk that can be influenced by social determinants and occupational exposures. Therefore, interventions that promote hydration practices, equitable protections, and healthcare access—particularly for Hispanic and less educated workers may reduce fatigue and related safety risks in this vulnerable workforce.

### Regional variation in measured health outcomes

Our adjusted multivariate analyses showed significant regional differences in the measured hydration-related health outcomes. Compared to the Northeast region, construction workers residing in the Midwest, Southern, and West regions had significantly lower odds of reporting back pain; the Midwest had slightly lower odds of reporting better general health (β = −0.13, *p* = 0.023), while associations for the Southern and West were weaker and not significant. These findings underscore how workers’ geographical location shapes their health, which may reflect regional differences in socioeconomic conditions, healthcare infrastructure, or labor practices. Regional differences were generally not statistically significant for fatigue, cognitive difficulty, and injury that resulted in missed workdays, though the West showed marginally lower injury odds (β = −0.515, *p* = 0.05).

### Public health and policy implications

The consistency of our findings strengthens the validity of the observed associations between regions and the measured health outcomes. These underscore the need for a comprehensive targeted national occupational health policy and regulation, including hydration practice promotion, targeted injury prevention strategies that protects all vulnerable workers from heat strain effects irrespective of their racial groups, especially during summer when workers are mostly vulnerable to extreme heat exposure with limited proper hydration practice, and detailed approaches to address cognitive and musculoskeletal health among aging and male construction workers. Also, considering the ongoing increasing ambient temperature and policy changes such as prohibiting/removal of water breaks for construction workers in Texas and Florida, two states in the Southern region, the need for close monitoring and evaluation of the potential effects of the removed water breaks on workers’ health outcomes, particularly hydration-related health outcomes due to workers extreme heat exposure vulnerability is essential in this region and across all states as, climate change an unsafe natural environmental conditions affects other states in the U.S. Further studies should assess the effects of the removed water break policies in Texas and Florida on construction workers’ reported hydration-related health outcomes, integrating workers’ occupational heat exposure and hydration practices. Furthermore, while this study identified the association among USA construction workers, it is worth noting that the findings reported in this study might have underrepresented the association, as approximately 13–15% of national construction workers are undocumented, with about 50% working in Texas, and might not have been captured in the NHIS dataset we analyzed. Therefore, we strongly recommend improving access to healthcare and injury reporting for uninsured and undocumented workers, which could help reduce underreporting and improve occupational surveillance.

## Conclusion

Construction workers’ age, educational attainment, race, BMI, marital and insurance status, shape workers’ risks of poor general health, severe back pain, cognitive difficulty, injury, and fatigue. Lower educational attainment, including less than HS, HS/GED, and some college degree, and obesity, increased workers’ vulnerability to the hydration-related health outcomes. The racial disparities reflect inequities in workers’ occupational exposures and resources. Importantly, the observed regional differences reflect how local work environments and climate conditions may shape workers’ hydration practices and the reported health outcomes, as workers in hotter regions may face greater risks of dehydration and fatigue if workplace protections are inadequate. These findings emphasize the intersection of social and geographic factors with hydration, underscoring the need for regionally tailored interventions and evaluating the effects of the recently removed water breaks in Texas and Florida. Also, strengthening hydration practices within broader occupational health strategies is essential to reduce disparities and protect construction workers’ health.

## Data Availability

The data is publicly available and can be accessed at https://www.cdc.gov/nchs/nhis/documentation/?CDC_AAref_Val=https://www.cdc.gov/nchs/nhis/data-questionnaires-documentation.htm.
