# Supplementary material for: Work- and hydration-related health outcomes prevalence among USA construction workers: evidence from the national survey
Source: Front Public Health. 2026 Jan 14;13:1721825. doi: 10.3389/fpubh.2025.1721825 (PMC12847283; doi:10.3389/fpubh.2025.1721825)
Supplement: Supplementary file 1 [file Table_1.DOCX]

**Measured health outcomes distribution by region**

1. ***General Health***

| **Region** | **Good health (%)** | **Moderate health (%)** | **Poor health (%)** |
| --- | --- | --- | --- |
| Northeast | 27.7 | 63.9 | 8.32 |
| Midwest | 22.7 | 68.43 | 8.86 |
| Southern | 25.7 | 64.85 | 9.45 |
| West | 26.0 | 65.19 | 8.78 |

**Note:** The general health 5-point scale was converted to a 3-point scale, with excellent changed to “Good health”, very good, and good merged and called “Moderate health”, while the fair and poor merged and called “Poor health”.

1. ***Back Pain***

| **Region** | **No (%)** | **Yes (%)** |
| --- | --- | --- |
| Northeast | 34.7 | 65.3 |
| Midwest | 30.7 | 69.3 |
| Southern | 32.6 | 67.4 |
| West | 31.2 | 68.6 |

1. ***Fatigue***

| **Region** | **No (%)** | **Yes (%)** |
| --- | --- | --- |
| Northeast | 64.96 | 35.03 |
| Midwest | 69.85 | 30.15 |
| Southern | 78.69 | 21.31 |
| West | 63.55 | 36.44 |

1. ***Cognition difficulty***

| **Region** | **No (%)** | **Yes (%)** |
| --- | --- | --- |
| Northeast | 85.89 | 14.11 |
| Midwest | 82.76 | 17.24 |
| Southern | 84.40 | 15.60 |
| West | 84.47 | 15.54 |

1. ***Injury***

| \| **Region** \| **No (%)** \| **Yes (%)** \| \| --- \| --- \| --- \| \| Northeast \| 56.46 \| 43.53 \| \| Midwest \| 59.14 \| 40.86 \| \| Southern \| 58.70 \| 41.30 \| \| West \| 68.47 \| 31.53 \| |  |  |
| --- | --- | --- | --- | --- | --- | --- | --- | --- | --- | --- | --- | --- | --- | --- | --- | --- | --- |
